# Regulatory Compliant Tissue-Engineered Human Corneal Endothelial Grafts Restore Corneal Function of Rabbits with Bullous Keratopathy

**DOI:** 10.1038/s41598-017-14723-z

**Published:** 2017-10-26

**Authors:** Gary S. L. Peh, Heng-Pei Ang, Chan N. Lwin, Khadijah Adnan, Benjamin L. George, Xin-Yi Seah, Shu-Jun Lin, Maninder Bhogal, Yu-Chi Liu, Donald T. Tan, Jodhbir S. Mehta

**Affiliations:** 1 Tissue Engineering and Stem Cell Group, Singapore Eye Research Institute, Singapore, Singapore; 2 Duke-NUS Graduate Medical School, Singapore, Singapore; 3 Singapore National Eye Centre, Singapore, Singapore; 4 School of Material Science and Engineering, Nanyang Technological University, Singapore, Singapore; 5 Yong Loo Lin School of Medicine, National University of Singapore, Singapore, Singapore; 6 Department of Corneal and External Disease, Moorfields Eye Hospital, London, UK

## Abstract

Corneal transplantation is the only treatment available to restore vision for individuals with blindness due to corneal endothelial dysfunction. However, severe shortage of available donor corneas remains a global challenge. Functional regulatory compliant tissue-engineered corneal endothelial graft substitute can alleviate this reliance on cadaveric corneal graft material. Here, isolated primary human corneal endothelial cells (CEnCs) propagated using a dual media approach refined towards regulatory compliance showed expression of markers indicative of the human corneal endothelium, and can be tissue-engineered onto thin corneal stromal carriers. Both cellular function and clinical adaptability was demonstrated in a pre-clinical rabbit model of bullous keratopathy using a tissue-engineered endothelial keratoplasty (TE-EK) approach, adapted from routine endothelial keratoplasty procedure for corneal transplantation in human patients. Cornea thickness of rabbits receiving TE-EK graft gradually reduced over the first two weeks, and completely recovered to a thickness of approximately 400 µm by the third week of transplantation, whereas corneas of control rabbits remained significantly thicker over 1,000 µm (*p* < 0.05) throughout the course of the study. This study showed convincing evidence of the adaptability of the propagated CEnCs and their functionality via a TE-EK approach, which holds great promises in translating the use of cultured CEnCs into the clinic.

## Introduction

A transparent cornea is essential for clear unobstructed vision and the corneal endothelium (CE) plays a critical role in maintaining corneal deturgescence, keeping the cornea clear^1,2^. The dynamic control of corneal hydration functions through a “leaky barrier and ionic pump” mechanism, which involves passive nutrient uptake from within the anterior chamber (AC) into the corneal stroma, as well as the maintenance of an ionic osmotic gradient that enables movement of excess fluid back into the AC^3–5^. Within the eye, the human CE is not known to undergo active cellular regeneration to replace damaged or dead corneal endothelial cells (CEnCs)^6^. Hence, in the event of an acute physical/surgical trauma or degenerative damage to the CE due to hereditary diseases, the reduction in corneal endothelial cell density below a critical threshold will diminish its functional efficacy, resulting in the accumulation of excess fluid build-up within the stroma. As such, a cascade of pathophysiological outcomes will ensue, starting with the edema of the stromal layer, causing corneal clouding, leading to a gradual loss of visual acuity over time, and will ultimately result in corneal blindness. Although the vision of these patients can be restored through a corneal transplant, a severe shortage of suitable donor graft material remains a pressing global issue^5^. A recent survey revealed that approximately 185,000 cases of corneal transplantation were performed in 116 countries in 2012 alone, but a conservative estimate made in the same reports puts demand for corneal transplantation at around 12.7 million^7^, providing the heightened impetus for the development of alternate treatment strategies through scalable cell-based regenerative therapeutics.

The human CE is non-regenerative within the eye, as the CEnCs are locked in a quiescent non-proliferative G_1_ phase of cell cycle^6,8^. However, these CEnCs can be isolated and grown within a conducive *in vitro* environment, as shown by Baum and colleagues over three decades ago^9^. Since that first report, and with better understanding of the cellular biology of primary human CEnCs, the culture of these unique cells has improved significantly over time^5,10^. The current approaches to propagating primary human CEnCs, as described by various laboratories around the world, differ vastly in terms of the formulation of the culture media used^11–15^. Recent studies within the field have been driven towards a common objective, targeting improvements to the expansion of primary human CEnCs. The scope of these studies ranged from protecting CEnCs against oxidative DNA damage^14^; selectively activating specific signaling pathway to modulate contact inhibition of CEnCs^16–18^; specific use of signaling molecules and/or inhibitors to prevent fibroblastic transformation of expanded CEnCs^12^; or to enhance the growth dynamics of CEnCs in culture^19–21^. However, most if not all of these reported methodologies of cultured human CEnCs are established using research-grade reagents or materials that were animal-derived and/or not well-defined^10^. For example, the use of an extracellular matrix (ECM) coating has been shown to significantly increase the adherence of human CEnCs onto cell-culture vessel^22–24^. One of the more popularly used ECM is the proprietary “FNC” coating mixture, containing a blend of bovine serum albumin, bovine collagen, and bovine fibronectin, which makes such reagent both animal-derived as well as undefined. Due to the potential risks of xeno-contamination, as well as the possible transfers of infectious pathogens, use of human CEnCs that were not propagated under good manufacturing practices (GMP) conditions in future clinical trials and clinically oriented cell-based therapeutics is not ideal. Achieving GMP compliance for any cell-based therapeutics must be done following strict regulatory guidelines as defined by the local regulatory body where the cellular therapy is being developed^10^. It is not a trivial process, and can be an arduous endeavor, as many regulatory hurdles must be satisfied. While regulatory guidelines will most certainly differ between regions, the underlying goal is to ensure both safety and quality of the developed cell-based therapeutics^10^.

We have described an approach for the isolation and propagation of primary human CEnCs using a robust dual media culture system, where the isolated CEnCs were cultured in a proliferative medium until they are near confluence before being switched into a maintenance medium^11^. In this present study, we first describe the refinement of the dual media approach of propagating human CEnCs towards a GMP-compliant system, using suitable GMP-grade replacements in place of research-grade and/or ill-defined reagents. The adaptation of key processes such as cellular digestion and dissociation, cellular adherence onto ECM, general growth dynamics, as well as cryo-preservation were systematically optimized and assessed modularly, to show that the changes from the research-grade reagents currently used, to GMP alternatives resulted in comparable or improved outcomes. All the finalized reagent changes were subsequently incorporated into an all-inclusive GMP-aligned culture system and CEnCs propagated using this GMP-aligned culture system (CEnCs_(GMP)_) were comparatively characterized for their expression of markers indicative of human CE at both the gene level using quantitative polymerase chain reaction (PCR) and their marker expressions using immuno-florescence. The expanded human CEnCs_(GMP)_ were also genetically assessed for karyotypic instability at the third passage. Finally, in order to show that the use of expanded human CEnCs_(GMP)_ is a viable therapeutic option, we assessed its functional capacity using a proof-of-concept tissue-engineering approach within a rabbit model of bullous keratopathy, where the propagated CEnCs_(GMP)_ were seeded onto a thin decellularised stromal lenticule with intact Descemet’s membrane (DM). Thus from start to finish, this study shows the robustness and adaptability of propagating human CEnCs using the dual media approach, and most importantly, provides an insight into the functional translation of tissue-engineered graft material generated using these CEnCs for the replacement of a damaged CE layer through current corneal transplant surgical technique.

## Results

### Digestion of Descemet’s membrane and dissociation of CEnCs

**Figure 1:**
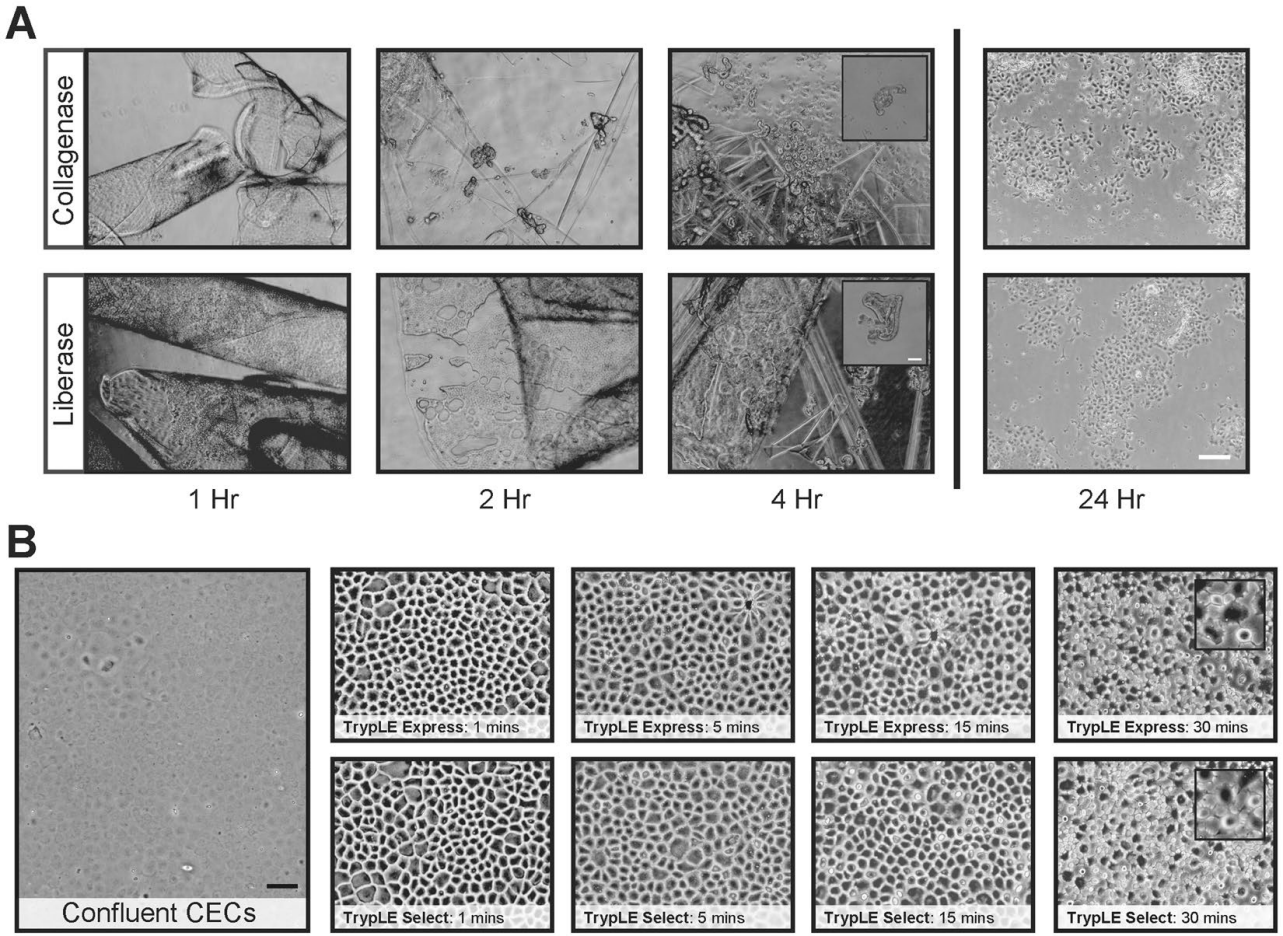
Descemet’s membrane complex digestion and corneal endothelial cells dissociation. (**A**) Representative micrographs of donor-matched corneal endothelium/DM complex digested in both Collagenase and Liberase at 1, 2, and 4 hours; as well as at 24 hours following plating of isolated cells. Scale bar: 200 µm. Scale bar of insert at 4 hours: 100 µm. (**B**) Images of confluent culture of human CEnCs subjected to TrypLE Express and TrypLE Select at 1, 5, 15 and 30 minutes. Scale bar: 100 µm. Insert box at 30 minutes: 100 µm.

Following initial evaluation of Liberase to obtain the optimal working concentration for the digestion of the DM to release the human CEnC-clusters, we found it to be comparable to that of the more commonly used research-grade Collagenase over a period of 4 hours (Fig. 1A; n = 4). The overall cellular yield per cornea, 24 hours post-attachment, as assessed over 3 separate isolations between Liberase and Collagenase were also comparable (Fig. 1A; n = 3). Our culture approach requires an additional step of cellular dissociation during initial isolation, to dissociate released human CEnC-clusters into smaller clumps and single cells, as well as during the normal course of culture to dissociate the adherent CEnCs from the culture surface during passaging. Due to constraints of insufficient material and ease of comparative assessment, observations for this comparison were made during passage, following cellular expansion only, and not during initial cell isolation. As expected, the rate of dissociation as assessed by the rounding up of the confluent CEnCs at the various time-point of observations between the two TrypLE reagents were comparable (Fig. 1B; n = 3).

### Cellular attachment of CEnCs – an assessment of extracellular matrices

**Figure 2:**
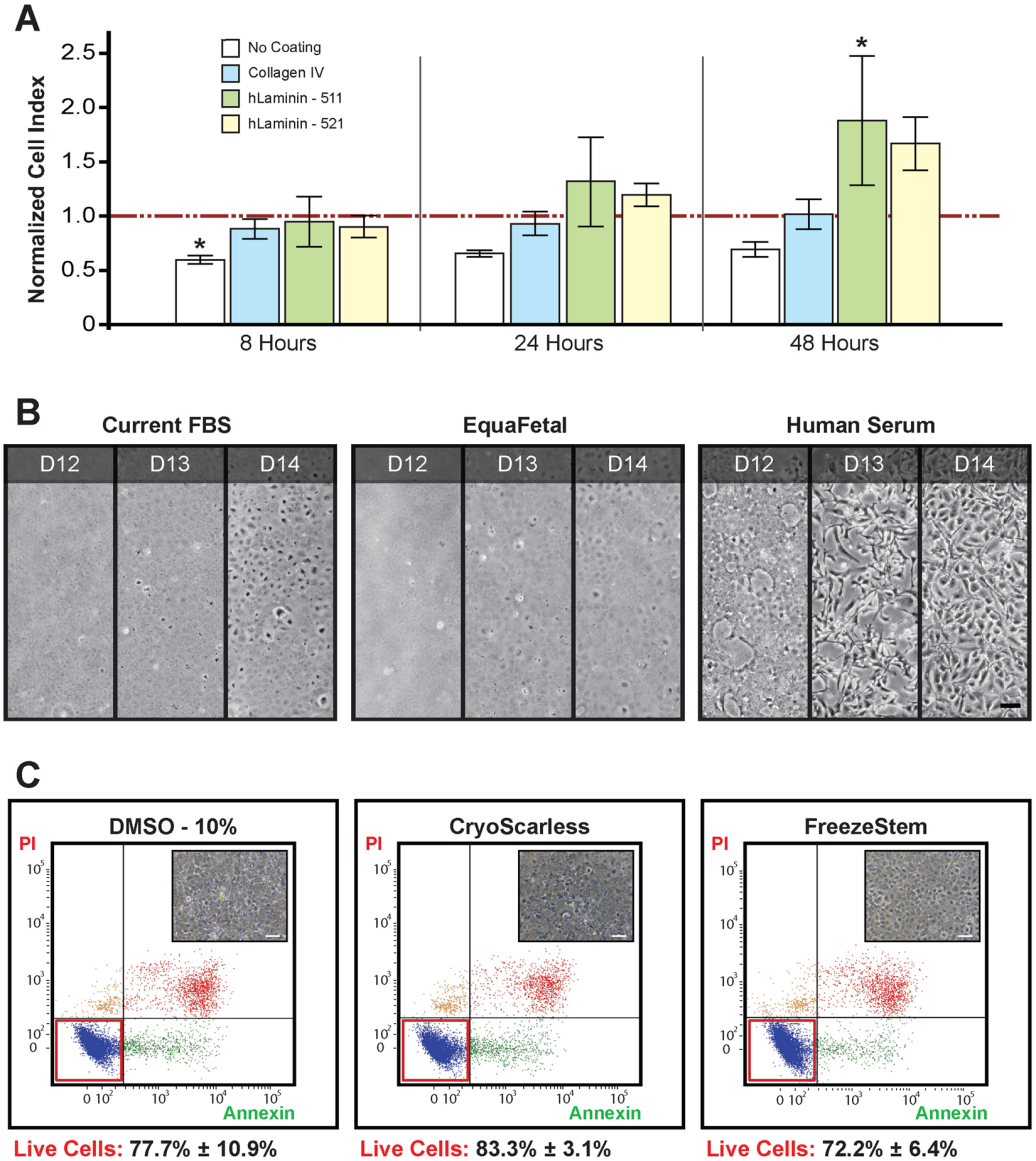
Comparative assessments of cell culture reagents. (**A**) Human CEnCs were seeded onto wells that were either pre-coated for FNC coating, human Collagen IV (blue), hLaminin-511 (green) or hLaminin-521 (yellow), or non-coated (white) as a negative control and analyzed for up to 48 hours using the xCELLigence system. Normalized impedance readings of CEnCs were extracted at 8, 24, and 48 hours. Cell index was normalized to 1.0 (red line) to cells seeded on FNC-coated wells for each of the time points. Statistical significance were detected between the impedance index of CEnCs seeded onto control vs FNC-coat wells (n = 4, *p < 0.05) at 8 hours, as well as between CEnCs seeded onto hLaminin-511 vs FNC-coated wells (n = 4, *p < 0.05). (**B**) Representative images showing the morphology of 3 confluent cultures of donor-matched human CEnCs grown in regular FBS, Equafetal, or commercially obtained HS. Scale bar: 100 µm. (**C**) Flow cytometric dot plots of overall percentage live cells (red box), as revealed by the Annexin V detection kit following cryo-preservation in current freezing solution of M5-Endo containing 10% DMSO, CryoScarless, and FREEZEStem. Insert within each condition showed morphology of cryo-preserved CEnCs that were revived directly into a high density seeding following cryo-preservation in its respective freezing medium. Scale bar: 100 µm.

We have previously shown that the use of FNC as an ECM coating on the culture surface greatly improves the attachment of human CEnCs^11,23^. Here, using FNC-coating as the gold standard for comparison (red-dotted line; Fig. 2A), we evaluated three ECM coatings as potential replacements for FNC: human Collagen IV, recombinant hLaminin-511, and hLaminin-521. Negative control was cells attaching on surfaces with no ECM coating. It should be noted here that hLaminin-511 and hLaminin-521 were identified from an initial screen, which included five other laminin isoforms: hLaminin-111, hLaminin-211, hLaminin-332, hLaminin-411, and hLaminin-421 (results not shown). Cellular attachment of human CEnCs was assessed using xCelligence comparing FNC-coating, hLaminin-511, hLaminin-521, and Collagen IV, all of which showed comparable attachment profile at 8 hours and 24 hours, with a notable but non-significant increase for CEnCs adhering in hLaminin-511 at 24 hours (Fig. 2A; n = 4). At 48 hours, the adherence of CEnCs in Collagen IV remained comparable to FNC-coating, whereas adherence of CEnCs cultured on hLaminin-521 also showed a notable but non-significant increase over cells on FNC-coating (Fig. 2A; *p* = 0.186). Interestingly, at the same time-point of 48 hours, the adherence of CEnCs cultured onto hLaminin-511 achieved statistical significance based on the observed impedance index when compared to cells cultured on FNC (Fig. 2A; *p < 0.05).

### Effect of sera on growth dynamics of CEnCs

The evaluation of the growth dynamics of human CEnCs cultured in M4 supplemented with different serum was assessed in an independent series of freshly isolated CEnCs (n = 5). To prevent exposure to any serum during the isolation process, CEnCs were first isolated using serum-free enzymatic digestion – Liberase, followed by a shorter exposure to TrypLE Select, as described earlier. Our results showed that CEnC-cultures grown to confluence in both FBS- and EquaFetal-supplemented medium were comparable in terms of their cellular morphology between donors (Fig. 2B). However, donor-matched cultures expanded in HS supplemented medium grew inconsistently, resulted in cultures that were morphologically heterogeneous, with cells that were not as tightly packed in some cultures, and fibroblastic-like in others (Fig. 2B). Further morphometric analysis of two randomly selected donor-matched CEnCs, showed that CEnCs grown in HS supplemented medium were significantly larger in size (Supplementary Fig. S1; *p < 0.05), and cells that were significantly less circular (Supplementary Fig. S1; *p < 0.05). Coefficient of variance evaluation also showed that CEnCs grown in HS supplemented medium were more variable in both cell size and cell circularity when compared to CEnCs grown in medium supplemented with either of the other two sera (Supplementary Fig. S1).

### Cryo-preservation of CEnCs

Primary CEnCs cryo-preserved in 10% DMSO were compared to cells that were cryo-preserved in two other DMSO-free cryo-preservation solutions, Cryoscarless and FREEZEstem (n = 4). Half of the post-thawed cells were immediately labelled with the live-dead assay and assessed for total percentage viable cells via flow cytometry. Here, we found that approximately 77.7% ± 10.9% of the CEnCs cryo-preserved in 10% DMSO survived the thawing process, whereas cell survival of CEnCs cryo-preserved in Cryoscarless were marginally better, but not statistically significant, with 83.3% ± 3.1% of viable cells. For cells cryo-preserved in FREEZEstem, approximately 72.2% ± 6.4% of the cells survived (Fig. 2C). Nevertheless, thawed primary CEnCs cryo-preserved in all three cryogenic preservation solutions could be successfully revived, and adhered to form a homogeneous monolayer of compact polygonal/hexagonal cell layers, as assessed following at least 2 days of culture in M5-Endo medium (Fig. 2C, see respective insert). As CEnCs cryo-preserved in Cryoscarless showed marginally better cellular survival over a short-term period of one week, we evaluated the long-term survival of CEnCs preserved in Cryoscarless for 24 months and found that cell survival of these CEnCs to be between 66.3% to 71.8%.

### Characterization of CEnCs_(GMP)_ cultured to the third passage

**Figure 3:**
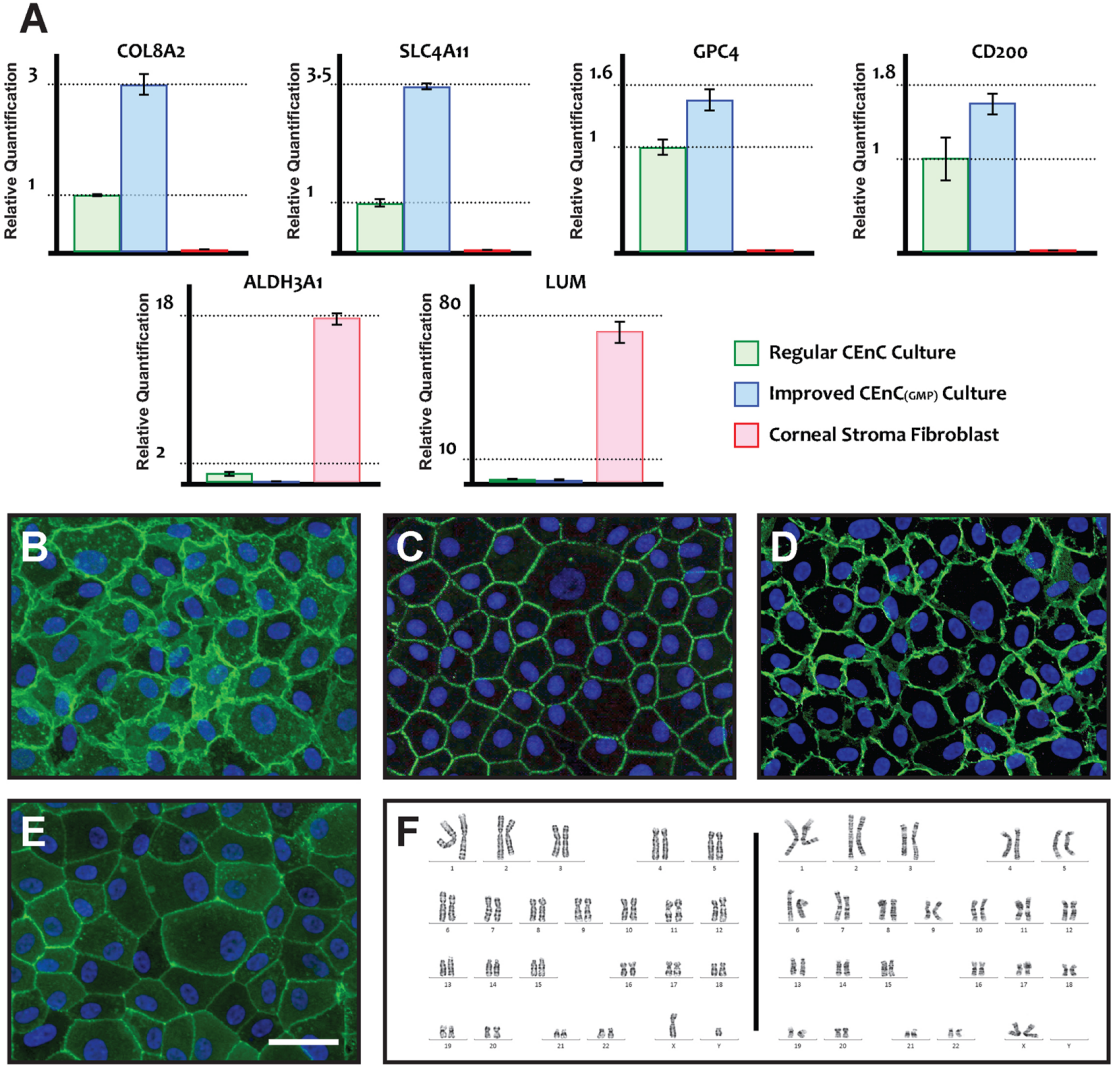
Characterization of corneal endothelial cells cultured to the third passage using refined dual media culture system. (**A**) Quantitative RT-PCR of mRNA extracted from confluent cultures of human CEnCs grown using regular dual medium (green), human CEnCs_(GMP)_ propagated using the refined dual medium (blue), and expanded human corneal fibroblast (red) were performed. Relative quantification values of each genes were calculated with *GAPDH* set as the internal control, and respective gene expression of *COL8A2*, *SLC4A11*, *GPC4*, *CD200*, *ALDH3A1*, *LUM* were calculated relative to human CEnCs from the regular culture. It should be noted that human CEnCs used for the gene expression study were non-donor matched but were both at the third passage. Primary human CEnCs_(GMP)_ were cultured to the third passaged and characterized for their expression of (**B**) Na^+^K^+^ATPase, (**C**) ZO-1, and cell surface markers (**D**) CD166; TAG-1A3 and (**E**) PRDX-6; TAG-2A12 by immunocytochemistry. Scale bar: 100 µm. (**F**) Representative karyograms of chromosomal spreads of CEnCs_(GMP)_ propagated to the third passage from a male donor (46, XY; left) and a female donor (46, XX; right).

Gene expression analysis showed that corneal endothelial-associated genes, *COL8A2* ^25^, *SLC4A11* ^25^, *GPC4* ^26^, and *CD200* ^26^ were expressed by CEnCs grown and expanded in both culture formulations, and not in corneal stroma fibroblast (Fig. 3A). Conversely, neither of the expanded CEnCs expressed *ALDH3A1* ^27^ nor *LUM* ^28^, in comparison to high level of expression detected in stromal fibroblasts control (Fig. 3A). Human CEnCs_(GMP)_ propagated to the third passage (n = 3) seeded at a density of at least 2,000 cells per mm^2^ consistently formed a homogeneous monolayer of cells, and expressed characteristic markers of the CE such as the pump-associated NA^+^/K^+^-ATPase (Fig. 3B), tight junction protein ZO-1 (Fig. 3C), as well as two recently reported cell-surface markers TAG-1A3^29^ – CD166 (Fig. 3D), and TAG-2A12^29^ – PRDX-6 (Fig. 3E). Chromosomal analysis were performed for human CEnCs_(GMP)_ cultured to the third passage from 6 donors. However, 2 sets of analysis were excluded due to insufficient metaphases counted. Out of the remaining 4 chromosomal analyses, 3 were of normal karyotype (Fig. 3F), and 1 showed evidence of chromosome 7 trisomy (Supplementary Fig. S2).

### Characterization of TE-EK grafts

Generated TE-EK grafts were characterized for their endothelial cell density at Day 14 and Day 28 following the seeding of the cultured CECs at 3,000 cells/mm^2^ onto the denuded DM/stroma lenticule. Results obtained at Day 14 showed that TE-EK graft 1 had 2,440 ± 207 cells/mm^2^ whilst TE-EK graft 2 had 2,780 ± 179 cells/mm^2^. At Day 28, corneal endothelial cell counts were 2,380 ± 192 cells/mm^2^ for TE-EK graft 1 and 2,680 ± 438 cells/mm^2^ for TE-EK graft 2 (Supplementary Fig. S3).

### Functionalization of CEnCs_(GMP)_ in a rabbit model of bullous keratopathy

**Figure 4:**
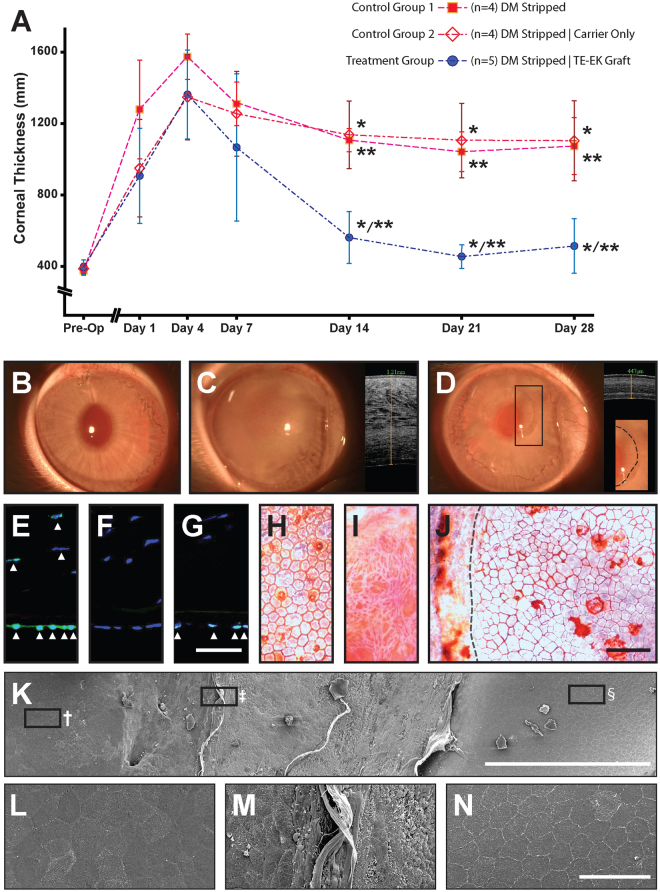
Functional assessment and characterization of the tissue-engineered corneal endothelial graft in a rabbit model of bullous keratopathy. (**A**) Graph summarizing the corneal thickness of the 3 groups of rabbits used in this study, including 2 control groups, post-operatively until 28 days following the surgeries. Controls were ‘DM stripped’ (n = 4; Control group 1) control where only the rabbit’s DM was removed and ‘DM stripped with carrier only’ (n = 4; Control group 2) control where the rabbit’s DM was removed, followed by transplantation of an empty carrier (without cells). Rabbits in the treatment group received TE-EK graft following removal of DM (n = 4). Corneal thickness of rabbits was comparable in all three groups as shown pre-operatively, and at the first, fourth and seventh day following surgery. The corneas of rabbits receiving the TE-EK graft were significantly thinner when assessed on Day 14 compared to control group 1 (*p < 0.05) and control group 2 (**p < 0.05). The statistical significance were maintained for assessments on Day 21, as well as on Day 28, where corneal thickness of rabbits with TE-EK grafts recovered completely to pre-operative corneal thickness of 459.3 µm ± 65.4 µm at Day 21, and 517.3 µm ± 151.3 µm at Day 28. (**B**) Representative slit-lamp image of corneal clarity were taken pre-operatively. Slit-lamp image and AS-OCT scan taken at Day 28 of (**C**) a rabbit in control group 2, showing an opaque cornea with a corneal thickness of approximately 1.21mm, and (**D**) a rabbit which received TE-EK graft with a clear central cornea and a corneal thickness of 447 µm. Distinctive areas with different degree of corneal clarity can be detected (see insert; dotted line). Staining of a human-specific nuclei antibody on (**E**) a human donor cornea as a positive control; (**F**) the rabbit cornea as a negative control; and on (**G**) an section of an excised cornea of rabbit receiving TE-EK graft Scale bar: 50 µm. Flat-mount Trypan Blue and Alizarin Red staining of (**H**) cornea of a normal rabbit; (**I**) stromal layer of cornea of rabbit from control group 1; and (**J**) cornea of rabbit receiving the TE-EK graft. Dotted lines demarcate the edge of the TE-EK graft and the underlying stroma layer Scale bar: 100 µm. (**K**) A panoramic SEM image of rabbit cornea showing the TE-EK graft, a zone of bare stroma and the rabbit own corneal endothelium. Scale bar: 400 µm. Magnified within (**K**): is (**L**) the TE-EK graft^†^; (**M**) the boundary between the TE-EK graft and the underlying stroma^‡^; and (**N**) the rabbit’s native corneal endothelium^§^. Scale bar: 50 µm.

For the *in vivo* studies, the corneal thickness of rabbits in both control group 1 ‘DM stripped’ control (n = 4) and control group 2 ‘DM stripped with carrier only’ control (n = 4), gradually increased over the first 4 days. Swelling of the corneas, as gauged by AS-OCT imaging, plateau by the fourth day to approximately 1,400 µm and remained above 1,000 µm for the duration of the 28 days observation period (Fig. 4A). In the treatment group (n = 5), the corneal thickness of the DM-stripped rabbit, transplanted with the TE-EK graft, increased in a similar manner as the 2 control groups for the initial 4 days. Thereafter, the corneas of rabbit receiving the TE-EK graft showed gradual thinning of the corneal stroma by the second week, which recovered close to the original pre-operative corneal thickness of 459.3 µm ± 65.4 µm on Day 21, and 517.3 µm ± 151.3 µm on Day 28 (Fig. 4A). These observations clearly showed the unequivocal rescue of the induced bullous keratopathy, and clear evidence of a functional corneal endothelium. Slit lamp photographs and AS-OCT scans taken at Day 28 before the rabbits were sacrificed clearly showed the differences between a representative control rabbit that received the carrier-only, where the corneal thickness remained at approximately 1210 µm (Fig. 4C) and a rabbit receiving the TE-EK graft, with a corneal thickness of 447 µm (Fig. 4D). Interestingly, a distinctive area of corneal clarity could be observed within the grafted region, corresponding to the relative size of the graft, where the peripheral region remained relatively hazy in comparison (Fig. 4D; insert).

### Characterization of the functional TE-EK grafts recovered from the excised corneas

We first assessed the specificity of the human-specific nuclei antibody and showed that it only labeled the nuclei of cells (arrowed) within the human cornea (Fig. 4E), but not the cells of the rabbit cornea (Fig. 4F). Indeed, only the cells of the corneal endothelial layer (arrowed) of the recovered TE-EK graft, but not the cells of the stromal layers, were positive for the human-specific nuclei antibody, indicating that these were indeed human cells (Fig. 4G). Histological analysis of excised corneas were performed using standard Trypan Blue and Alizarin Red staining. Labeled flat-mount cornea of normal rabbit showed a regular mosaic layer of homogenous hexagonal corneal endothelial cells (Fig. 4H), whereas only bare corneal stromal bed was observed in the corneas of rabbit in the ‘DM stripped’ group (Fig. 4I). Labeled flat-mount preparation of excised cornea with the TE-EK graft showed a cellular layer with heterogeneously shaped cells that were irregularly polygonal (Fig. 4J), indicative of a tissue-engineered construct. It should be noted that the dotted lines demarcate the edge of the TE-EK graft (Fig. 4J). Representative SEM images of the excised cornea with the TE-EK graft showed 3 distinctive regions, with an acellular bare stromal region that lies between the TE-EK graft and the native rabbit corneal endothelial cells (Fig. 4K). No evidence of any cell migration was observed. Higher magnification SEM images showed the heterogeneously shaped human CEnCs_(GMP)_ on the TE-EK graft^†^ (Fig. 4L), the edge of the TE-EK graft and the underlying stromal bed^‡^ (Fig. 4M), and the homogenous mosaic pattern of the rabbit’s own corneal endothelium^ϕ^ (Fig. 4N).

## Discussion

Corneal transplantation is one of the most commonly performed transplant surgeries in the world, restoring vision to individuals afflicted by corneal blindness^30^. Even though 184,576 corneal transplants were performed in 2012 worldwide^7^, there is a global shortage of donor corneas far in excess of this number, and the demand for corneal transplantation will undoubtedly increase with an aging global population^31^. A conservative estimate puts the size of global waiting-list for corneal transplantation at 12.7 million, with at least 39% of these due to a dysfunctional corneal endothelium^7^. Based on these numbers, it is clearly evident that current donor cornea recovery efforts for corneal transplantation are not sustainable, and this gap will continuously widen, exponentially. As such there is a heightened impetus for the development of alternative strategies for the treatment of reversible corneal blindness, and two potential clinical treatment modalities using cultured primary human CEnCs, by intra-cameral cell-injection or via a cell and tissue engineering cell-carrier approach has been proposed^10,32^.

The development of any cell-based therapeutics towards the aim of alleviating the heavy dependency of donor cornea for EK surgeries will require the capacity to propagate desired *bona fide* primary human CEnCs with relative consistency and robustness. Building upon our previous reports on the propagation of primary human CEnCs using a dual media approach^11,19^, we identified the components that were either of research-grade, or not well-defined in that media formulations, and found equivalent replacements that were either of clinical grade, or were well-characterized, and GMP-compliant. By reducing as much of the undefined and/or animal-derived reagents used within the current culture system, potential threats of pathogenic transmission and immunological reactions following transplantation will naturally be lowered^33^. There is a necessity in moving towards a GMP compliant culture system, which will eventually be assessed by country-specific local regulatory body in terms of the safety of using cultured primary CEnCs for future human clinical trial.

Initial comparative studies between current research-grade reagents against its respective GMP-alternatives were performed modularly for each of the key processes (Supplementary Fig. S4), and must show outcomes that were as good, if not better, to be included in the final improved GMP-aligned formulation for the culture of human CEnCs_(GMP)_. The digestion of the DM to release the donor corneal endothelium and the subsequent dissociation of the released CEnC-clusters into smaller clumps are vital initiation steps to establish a successful culture of CEnCs^11,23^. Digestion of the DM using Liberase TH, a blend of both Collagenase I/II with a high concentration of Thermolysin, were comparable to using regular Type I Collagenase, and cell yield following cellular attachment were also comparable (Fig. 1A). More importantly, the incorporation of Liberase TH into this study is its availability of clinical GMP grade Liberase TH of the same formulation, beneficial for the transition to future clinical translational work. Dissociation of CEnCs were assessed by treating confluent donor-matched cultures of primary CEnCs with either TrypLE Express or TrypLE Select, and were found to be comparable (Fig. 1B), which was not surprising as both TrypLE reagents are made from highly purified, recombinant cell-dissociation enzymes free from animal-derived components. The difference lies in the production of TrypLE Select, which is manufactured on dedicated animal origin-free equipment. On the use of ECM, we and others have previously shown that although not critical, the use of a suitable ECM coating can significant improve the attachment of primary CEnCs^22,34^. In this study, we further showed that the use of Collagen IV derived from human placenta as ECM were comparable to FNC-coated vessels for the cellular attachment of primary human CEnCs (Fig. 2A). We also found that both coatings of hLaminin-511 and hLmaninin-521 provided even better outcomes for the attachment of human CEnCs (Fig. 2A) confirming a recent study that both hLaminin-511 and hLaminin-521 enhance the adhesion of CEnCs^35^. However, human Collagen IV was selected for the rest of this study, as it was significantly less expensive by at least fifteen times when compared to either hLaminin-511 or hLaminin-521.

Supplementation of basal culture media with serum is essential for the propagation of human CEnCs as it stimulate cell proliferation^23,36^. However, the very usage of serum also brings with it much undesirable uncertainties due to the complex mixtures of ill-defined constituents^37^, as well as significant batch-to-batch variability both quantitatively as well as qualitatively in terms of the composition of its constituent^38^. Serum can also be a potential source of microbial contaminants including fungi, bacteria, viruses or prions^39^. We had previously tried to culture primary CEnCs within a serum-free environment, replacing FBS with a KnockOut Serum Replacer (KO-SR), or removing FBS altogether, but isolated primary CEnCs were unable to thrive in those conditions (results not shown). Hence, with some form of serum being necessary, we continued its use, and for this study compared our current FBS with sera from two other sources, another bovine serum, Equafetal, and commercially available HS, reported to support the culture of primary CEnCs^40,41^. Interestingly, in our comparisons, primary CEnCs cultured in either of the two bovine sera were comparable as opposed to the morphological and proliferative inconsistency observed for CEnCs from all 5 donors that were propagated in HS supplemented medium (Fig. 2B). Further morphometric analysis of two randomly selected donor-matched CEnCs grown in the three sera revealed that CEnCs grown in HS supplemented medium to be significantly larger in sizes (Supplementary Fig. S1; *p < 0.05), and less circular in shape (Supplementary Fig. S1; *p < 0.05), indicating that these CEnCs were generally more elongated and fibroblast-like. It should be noted here that even though Equafetal is currently being used in several clinical trials across the globe, the idea of using HS has a much greater appeal over the use of animal serum as Equafetal does constitute the main sources of animal derived components within the culture system.

Being able to cryo-preserve primary human CEnCs is important as it potentially enables the distribution of propagated CEnCs to secondary sites for clinical use, as well as mitigating the time-sensitive aspect currently present with donor tissue. Previously described approaches generally used freezing solution containing 10% DMSO within a serum-containing medium for the cryo-preservation of CEnCs^19,40^. Despite being frequently used as a cryo-protectant, DMSO is also known to have multiple effects on cellular function, affecting cell cycle, as well as apoptosis in certain cell types^42^, and is a known cell-differentiating agent^43^. Comparing the post-thaw cell viability of primary CEnCs following cryo-preservation in two DMSO-free and serum-free cryo-preservation medium, Cryoscarless and FREEZEstem, to conventional DMSO containing cryo-preservation medium showed similar total live cell count as analyzed by flow cytometry. Although no statistical significance was observed in the cryo-preservation of 4 separate donor derived primary CEnCs, overall cell survival of CEnCs cryo-preserved in Cryoscarless were highest at 83.3% ± 3.1% over both conventional 10% DMSO at 77.7% ± 10.9% and FREEZEStem at 72.2% ± 6.4% (Fig. 2C). More importantly, we showed here that cryo-preserved CEnCs could be recovered successfully following an extended period of cryo-preservation of at least 24 months, and can also be revived directly into a high density seeding to obtain CEnCs with relatively homogeneous morphology, suggesting that it may be possible to distribute these cryo-preserved cells worldwide for the purpose of corneal endothelial cell therapy. However, it appeared that the rate of cell survival of CEnCs differs between donors, and the clinical application of cryo-preserved CEnCs for transplantation in terms of the functionality of the cryo-preserved CEnCs will have to be evaluated and characterized further in a separate study.

Combining the selected GMP reagents, we propagated an independent series of CEnCs_(GMP)_ and showed that these cells maintained gene expressions indicative of the CE such as *COL8A2*, *SLC4A11*, *GPC4*, *CD200*, and do not express non-CE genes *ALDH3A1*, and *LUM* ^25–28^. These CEnCs_(GMP)_ also expresses classical CE markers including pump-associated NA^+^/K^+^-ATPase, tight junction protein ZO-1, as well as cell-surface markers CD166 (TAG-1A3), and PRDX-6 (TAG-2A12)^29^. In our studies, out of the 4 sets of CEnCs_(GMP)_ with at least 20 metaphases analyzed for their karyotype, 1 was found to contain chromosomal aberrations of chromosome 7 trisomy (47, XX, +7; Supplementary Fig. S2). The appearance of chromosomal abnormality in cultured human CEnCs was not unexpected as it has been previously described by Miyai and colleagues^44^, and more recently by Hamuro and colleagues^45^. In those reports, it appeared that donor age and frequency of aneuploidy can be positively correlated, especially for the loss of Y chromosomes^44,45^. In our analysis, we did not detect any loss of Y chromosome, but it should be noted that the donor corneas we procured for our study were relatively young in comparison, and the culture of CEnCs_(GMP)_ with a clone of trisomy 7 were the oldest (35-years old) among the 4 sets of CEnCs_(GMP)_ that were evaluated. However, one has to concede that the necessity to expand CEnCs_(GMP)_ for the very purpose of cellular therapy inevitably subject these cells to considerable level of cellular stress associated with processes involved in primary cell culture, such as cellular dissociation. Previous studies suggested that recurring aberrant karyotypes conferred survival and proliferation advantages to pluripotent stem cells in prolonged culture, allowed them to continue to self-renew and prevent spontaneous differentiation^46,47^, or possibly linking them to premalignant transformations^48^, and it is unknown if the karyotypic changes observed in our cultured cells are of a similar nature. To date, reports that described karyotype changes in cultured human CEnCs, including ours, appeared to be sporadic, and randomly occurring^44,45^. Hence, it is unclear if these occurrences of chromosomal aberration are due to unfavorable conditions during the life of the donors (environmental factors, UV exposures) or during propagation of the CEnCs (sub-optimal culture conditions), where chromosomal instability allows for rapid adaptation through advantages conferred by karyotypic diversity^49^; or are naturally occurring such as those seen in cells within the brain^50^ and liver^51^. Ultimately, whether such aneuploidy found within cultured human CEnCs is able to promote tumorigenesis may depend entirely on the loci where the sporadic aberration occurs, and should be the subject of future studies. Until such day, human CEnCs procured for the purpose of clinical therapies, where possible, should be obtained from corneas from younger donors where occurrences of karyotype changes in cultured human CEnCs are lower^44^.

It is imperative that the functionality of the cultured CEnCs_(GMP)_ be evaluated and we opted to assess the functional capacity of the cultured CEnCs_(GMP)_ in an rescue scenario within a rabbit model of bullous keratopathy. Several reports of injected cultured mammalian CEnCs have been described within rabbit^52,53^, feline^54^ and monkey^52,55^ animal model. Although the cell-injection approach has been deemed as a relatively simple and minimally invasive procedure, backed with early promising results^52^, conflicting outcomes have since arisen^54^. Conceptually, an injection of CEnCs may seem ideal therapeutically, however, there are several contentious issues. For example, following the injection of CEnCs into the aqueous AC, the subject/patient must be moved to adopt a “face-down” prone position so as to promote cell deposition by gravity. This position must be maintained for at least three hours to allow for cellular adherence of the injected CEnCs. The process of cell injection, followed by the subsequent adoption of a “face-down” position, within an aqueous environment, will inevitably displace the injected cells within the AC resulting in ectopic deposition of the injected cells^54^. Furthermore, the cell adherence efficiency of 40-50% of the injected CEnCs, even in the presence of Rho-associated kinase inhibitor Y-27632^55^, warrants much improvement in its attachment efficacy as this qualitatively affects and ultimately dictates the consistency of the eventual cell density of the treatment via cell-injection of CEnCs. In terms of safety of the cell-injection, long-term observational outcome for any potential adverse outcomes will be required, if indeed a fraction of the injected cells do get washed out via the aqueous flow. There is also concern, in terms of treatment consistency, of potentially losing some of the injected cells through leakage out from the needle track created for the injection of the cells. From a clinical standpoint, the success of the cell injection approach requires the presence of the recipient’s own DM following removal of the CE^52,53^. Hence, the cell injection approach may not be suitable for cases of advanced Fuchs dystrophy, one of the leading indication (39%) for corneal transplant surgery^7^, where the DM became inundated with drop-like excrescences known as guttata^56^, and must be removed to reduce visual aberrations. Due to these concerns, we decided to assess cellular functionality of the cultured CEnCs_(GMP)_ through a tissue-engineered carrier-based approach, which will enable the implantation of a corneal endothelial graft equivalent, engineered with a defined number of cells, similar to that of EK surgeries in the form of TE-EK. In fact, the proof-of-concept animal study was specifically designed and experimentally adapted, based on an established and widely used posterior lamellar graft procedure, endothelial keratoplasty (EK), also known as Descemet’s Stripping Automated Endothelial Keratoplasty (DSAEK)^57,58^, which is routinely performed on patients undergoing corneal transplantation. Following initial optimization of the surgical approach for rabbits, implantation of the TE-EK graft into the anterior chamber of the rabbit using the Endoglide^57^ was reproducible. More importantly, it showed the translatability of the TE-EK approach from a surgical standpoint. Furthermore, the need to show the robustness of TE-EK approach therapeutically is necessary as the very concept of transplanting cultivated CEnCs as a form of cell replacement therapeutic is not new^5^. This idea has first been described as early as 1979 in the transplantation of cultured bovine CEnCs onto denuded cat corneas^59^. For human CEnCs, cell sheet of cultured CEnCs was reported to be functional when transplanted into the eye of a rabbit through an adapted PK approach in 2005^60^. However, clinical translation has not been achieved to date, and this is likely due to the poor handle-ability of the cellular sheet generated.

In our study, corneas of rabbits receiving the TE-EK graft substitutes were able to re-establish the regulation of corneal hydration by the second week following surgery, even despite corneal swelling to over 1,000 micron by the fourth day following removal of the rabbit’s own corneal endothelium. By the third week, the rescued corneas were as thin and clear as they were pre-operatively, and the maintenance of corneal deturgescence was sustained until the end of the study at the fourth week (Fig. 4A–D). Both populations of control rabbits showed corneal thickness of over 1,000 micron throughout the length of the study (Fig. 4A). Evidence from the immunohistochemical staining of human nuclei (Fig. 4G), Alizarin red histology (Fig. 4J), as well as SEM of the recovered TE-EK graft (Fig. 4K–N) from the excised corneas convincingly showed that the functional recovery observed were conferred by the cultivated human CEnCs_(GMP)_ and not due to the migration of the rabbit’s own CEnCs re-establishing functional integrity. More importantly, the approach to surgically insert the TE-EK graft for transplantation, being similar to that of DSAEK, indicates that it is practical and clinically feasible, and will be further evaluated in a first-in-man clinical Phase 1 trial in the near future. However, the use of a denuded human stroma carrier with DM-intact serves only as a ‘proof-of-concept’ in the generation of the TE-EK graft to assess the functionality of propagated human CEnCs_(GMP)_. Future studies to identify a potential thin, transparent biosynthetic carrier as a scaffold carrier with similar mechanic properties as that of the thin stroma with DM-intact; cellular compatibility with cultured human CEnCs_(GMP)_; and most importantly, scalability within a GMP environment in currently underway^61^.

It should be noted that the TE-EK grafts generated in this study uses primary human CEnCs expanded to the first or second passage. Based on previous estimates, including the improvements made to the dual media expansion of CEnCs^11,19,23^, and taking into consideration the numbers of CEnCs obtainable from each donor corneas can be significantly variable, due to donor variation, CEnCs isolated from a single cornea expanded with the current method should be able to generate between 2.05 × 10^6^ cells to 5.4 × 10^6^ cells at the first passage, and approximately 4.4 × 10^6^ cells to 1.26 × 10^7^ cells at the second passage. Based on these projected numbers, there will be sufficient cells for CEnCs grown to the first passage to generate between 13 to 36 TE-EK grafts of 8 mm diameter with a seeding density of 3,000 cells/mm^2^; or between 29 to 84 TE-EK grafts with CEnCs grown to the second passage.

We have showed that the generated TE-EK grafts, seeded at a density of 3,000 cells/mm^2^ were able to maintain a relatively high corneal endothelial cell counts of between 2,380 ± 192 cells/mm^2^ and 2,680 ± 438 cells/mm^2^ over 28 days of assessment *in vitro* (Supplementary Fig. S3). Although the post-operative cell counts of the transplanted TE-EK graft *in vivo*, taken at the third week showed a lower cell count of 1,321 ± 23 cells/mm^2^, it is likely due to factors such as surgical trauma, comparable to that of complex clinical DSAEK cases in patients^62^, as well as potentially xeno-rejection.

In conclusion, to translate any cell-based therapeutics and research outcomes obtained from the laboratory towards a clinical setting requires GMP compliance to ensure both safety and quality of the proposed cell-based treatment. In this study, we showed that the dual media approach of propagating human CEnCs is robust in a way that it is adaptable to changes within its formulation towards GMP compliance, and results of the characterization studies of expanded CEnCs_(GMP)_ showed outcomes that are as good if not better than CEnCs cultured using the original dual media culture formulation. More importantly, convincing evidence of human CEnCs_(GMP)_ functionality, in the form of a proof-of-concept TE-EK surgery, was shown in an animal model of bullous keratopathy where complete reversal of corneal blindness were achieved by the third week following transplantation. Taken together, the translation of propagated CEnCs_(GMP)_ into the clinics via the TE-EK approach holds great promise.

## Methods

### Study design

This study was approved by Singhealth centralized institutional review board (2013/783/A). There are two key objectives to our study. The first was to show the robustness of the dual media approach in propagating primary human CEnCs, whereby after one cycle of culture formulation refinement towards regulatory compliance, continues to generate *bona fide* CEnCs. Next, was to demonstrate both the functionality of the tissue-engineered CEnCs through a carrier-based approach via TE-EK in a pre-clinical rabbit model of bullous keratopathy and its clinical practicality from a surgical standpoint. Primary human CEnCs were isolated from research-grade cadaver corneal tissues procured through Lions Eye Institute for Transplant and Research (Tampa, FL, USA) and Miracles in Sights (Winston-Salem, NC, USA), obtained with informed consent from the next of kin, and adhered to the principles outlined in the Declaration of Helsinki. Initial assessment through cellular morphology, and morphometric analysis, were followed by more detailed cell adhesion assay, flow cytometry, gene expression, immunocytochemistry, as well as chromosomal analysis. For the *in vivo* pre-clinical portion, 12 New Zealand white rabbits (*Oryctolagus cuniculus*; 2.5–3.5 kg, 3–6 months old) were used for this study. Their use, care and treatment strictly adhered to the regulation of the ARVO statement for the Use of Animals in Ophthalmic and Vision Research, and all experimental procedures were approved by the Institutional Animal Care and Use Committee of SingHealth, Singapore (2013/SHS/0872).

All *in vitro* analyses were performed on data collected with at least a minimum of *n = *3 biological replicates. For the pre-clinical *in vivo* study, functionality of each TE-EK graft was assessed for its capacity to re-establish corneal deturgescence, keeping the cornea clear following removal of host CE. Two control groups were included in this study alongside the rabbits receiving the TE-EK grafts: “DM Stripped” and “DM Stripped/Carrier Only” which will indicate the occurrence of or dictate the rate of any spontaneous recovery^63,64^ of the host CE layer if any. Slit lamp images were taken throughout the study, and corneal thickness were measured from anterior segment-optical coherence tomography (AS-OCT) scans of corneal cross-sections using Visante AS-OCT. At the 28-day end point of the *in vivo* study, tissue outcomes were evaluated by immunostains, histology, and scanning electron microscopy (SEM) for comparative assessment.

### Materials

Ham’s F12, Medium 199, Human Endothelial-SFM, fetal bovine serum (FBS), Dulbecco’s Phosphate-Buffered Saline (PBS), Insulin/Transferrin/Selenium (ITS), Collagenase Type I, TrypLE^TM^ Express (TE), TrypLE^TM^ Select (TS), gentamicin, amphotericin B, 5-ethynyl-29-deoxyuridine (EdU) incorporation Click-iT Alexa Fluor 488 cell proliferation assay kit, Fix and Perm (Medium A), penicillin and streptomycin were purchased from Life Technologies (Carlsbad, CA, USA). Dimethyl sulfoxide (DMSO), trypan blue (0.4%), alizarin red, paraformaldehyde (PFA), human serum (HS), Collagen IV from human placenta, ascorbic acid, and ascorbate-2-phosphate were purchased from Sigma (St. Louis, MO, USA). Recombinant basic fibroblast growth factor was bought from R&D Systems (bFGF, Minneapolis, MN, USA). Rho-associated, coiled-coil protein kinase inhibitor Y-27632 was brought from Miltenyi Biotec (Bergisch Gladbach, Germany). FNC coating mixture was obtained from United States Biologicals (Swampscott, MA, USA). Liberase TH was purchased from Roche (Mannhein, Germany). EquaFetal® was from Atlas Biologicals (Fort Collins, CO, USA). FREEZEstem cryo-preservation medium, LAMSCREEN^TM^, human recombinant laminin-511 and 521 were bought from BioLamina (Sundbyberg, Sweden). Finally, Cryoscarless cryo-preservation medium was from Funakoshi (Tokyo, Japan).

### Research-grade human corneoscleral tissues

All research-grade human cadaver corneal tissues procured for this study through Lions Eye Institute for Transplant and Research and Miracles in Sights were obtained with informed consent from the next of kin of all deceased donors regarding eye donation for research, and adhered to the principles outlined in the Declaration of Helsinki. A total of 35 pairs of donor corneal tissues ranged from 2 to 35 years old (Table 1) with endothelial cell count of at least 2,200 cells per mm^2^ were procured for the culture of human CEnCs. Corneoscleral tissues were preserved in Optisol-GS (Bausch & Lomb, Rochester, NY, USA) at 4 °C until they were processed, usually within 14 days of preservation. It should be noted that three of the donor pairs procured from this study were isolated at 16, 18, and 19 days following preservation. However, overall assessments of initial cellular yield, followed by its cellular morphology and expansion profile after isolation was deemed to be acceptable, and hence, used in this study. Another 11 corneas were procured for the creation of the DM/stroma lenticular discs (Supplementary Table S1) using a femtosecond laser system (see below).

**Table 1:** Donor information for culture of primary CEnCs.

Serial Number	Age	Sex	Days to Culture	Cell Count (OS/OD)	Cause of Death	Figure				
						1	2	3	4	S
01	31	M	7	2959/2899	Brain Bleed	●				
02	31	F	9	2985/3322	Acute Cardiac Crisis	●				
03	23	F	10	2451/2519	Acute Cardiac Crisis	●				
04	21	M	5	2817/2907	Subarachnoid hemorrhage	●				
05	18	M	12	2778/2890	Multi-Vehicle Accident	●				
06	19	F	9	2770/2755	SI-GSW-Head	●		●		
07	25	M	12	2425/2506	Cerebral Vascular Accident	●		●		
08	7	M	6	2941/3003	Trauma		●			
09	30	M	6	2950/3058	Multi-Vehicle Accident		●			
10	24	M	8	3003/3236	Multi-Vehicle Accident		●			
11	33	F	12	2825/2584	SI-GSW-Head		●			
12	14	M	12	3215/3021	Drowning		●			
13	31	F	10	2653/2725	Multi-Vehicle Accident	●	●			●
14	31	F	7	2222/2218	Overdose		●			●
15	33	M	8	2551/2326	Trauma		●			
16	24	F	6	2457/2481	Acute Cardiac Crisis		●			
17	9	M	10	3774/3846	Total Heart Block	●				
18	14	F	5	3115/3208	Cerebral Edema	●				
19	21	F	14	3472/3610	Pulmonary Embolism		●			
20	22	M	12	3115/3145	Blunt Traumatic Injuries		●			
21	14	M	18	3215/3021	Drowning		●			
22	28	F	7	2833/2950	Suicide		●			
23	20	F	10	2725/2538	Multi-Vehicle Accident			●		
24	26	F	8	3268/3279	Intracerebral Hemorrhage			●		
25	30	M	19	3185/2944	Overdose			●		
26	3	M	9	4082/3968	Drowning			●		
27	5	M	16	3534/3247	Multi-Vehicle Accident			●		
28	18	M	10	2410/2404	Multi-Vehicle Accident			●		
29	35	F	14	2513/2667	Pulmonary & Cerebral Edema			●		●
30	28	M	8	3106/3125	Overdose				●	
31	19	F	12	3175/2890	Craniocervical Dislocation				●	
32	24	F	8	2950/2865	SI-GSW-Head				●	
33	17	F	11	3571/3472	Hanging				●	
34	2	F	11	4000/4016	Embryonal Tumor					●
35	15	F	12	2809/2985	Multiple Blunt Force Injuries				●	●

Donor age ranged from 2 year-old to 35 year-old with a median age of 22 year old. Days taken from death of donor to the initiation of corneal endothelial cell culture ranged from 5 days to 19 days with a median of 10 days.

### Cell isolation and cell culture

Primary human CEnCs isolated from pairs of donor corneas were propagated using a dual media system as previously described^11,19^, with modifications to evaluate the various reagents that were assessed within this study (Supplementary Fig. S4). Briefly, CEnCs were isolated through an initial enzymatic DM-digestion for approximately 4 hours. Dislodged CEnC-clusters were then treated with TrypLE reagent for 5 minutes to further dissociate the clusters into smaller clumps. These were then washed twice before being seeded onto ECM-coated culture vessels to be established in cornea endothelial maintenance/stabilization medium (M5-Endo; Human Endothelial-SFM supplemented with 5% serum) overnight. Subsequently, CEnCs were cultured in a proliferative medium (M4-F99; Ham’s F12/M199, 5% serum, 20 μg/ml ascorbic acid, 1x ITS, and 10 ng/ml bFGF) to promote the proliferation of isolated CEnCs. It should be noted here that unless otherwise stated for specific comparative assessment, FBS were used as serum supplement. Once proliferating CEnCs become 80% to 90% confluent, the cells were then maintained in M5-Endo for at least two days before being sub-cultured via single-cell dissociation using TrypLE reagent. Dissociated CEnCs were plated at a seeding density of at least 1 × 10^4^ cells per cm^2^ (a split ratio of between 1:3 to 1:4 dependent of the final cell count before passaging), on pre-coated surfaces for further expansion, or at higher seeding densities as described for characterization. All cultures were incubated in a humidified atmosphere at 37°C and 5% CO_2_.

For the comparison of sera used in culture, the study was designed so that CEnCs from each independent biological pair were isolated in serum-free conditions before being separated equally into the three proliferative conditions where the base M4-F99 medium were reconstituted with the three different serum (n = 5). The three sera used in this study were: (1) FBS, as the gold standard control used in the original regular dual media culture as previously described^23,34^; (2) EquaFetal, a serum produced from animal that have been maintained on controlled diets and living conditions, used as a direct replacement for FBS, but more importantly is being used in clinical trials that have been accepted from the Food and Drug Administration (USA), Medicines and Healthcare products Regulatory Agency (UK), and Pharmaceuticals and Medical Devices Agency (Japan) (Brent Bearden, Vice President, Atlas Biologicals, personal communication, 2015); and (3) commercially available human serum.

### Cell adhesion assay

The adherence of CEnCs on various pre-coated surfaces evaluated in this study was assessed using xCelligence real-time cell analyzer (ACEA Bioscience, San Diego, CA, USA). Primary human CEnCs expanded to the second or third passage were dissociated into single cells and seeded at a density of 2.5 × 10^4^ cells per cm^2^ in each well of an E-Plate 96 (ACEA Biosciences, San Diego, CA, USA), and left to attach in M5-Endo medium. Wells were pre-coated with human Collagen IV (50 µg/mL), hLaminin-511 (50 µg/mL), or hLaminin-521 (50 µg/mL). Wells pre-coated with FNC coating mixture were positive controls, whereas wells with no coating were negative controls. The electrical impedance readings of overall cell adherence were recorded using the xCELLigence real time cell analyzer throughout the experiment for at least 48 hours.

### Cryo-preservation and cell survival assay

Primary human CEnCs at the third passage were used in the evaluation of DMSO-free cryo-preservation solutions: Cryoscarless and FREEZEstem, in comparison to current freezing medium made up of 10% DMSO in M5-Endo. Following TE dissociation, approximately 3 × 10^5^ donor-matched CEnCs were separately re-suspended in each of the three freezing medium, and dispensed into the respective cryovial (Nalgene, Thermo Fisher Scientific, Waltham, MA, USA), placed in a freezing container (Nalgene) containing isopropyl alcohol (Merck Millipore, Billerica, MA, USA), and left in −80 °C freezer overnight. The next day, cryovials were transferred and stored in liquid nitrogen for at least one week, and up to 28 months. In order to assess the overall viability of the donor-matched human CEnCs that were cryo-preserved in their respective freezing medium, cryovials were retrieved and thawed in a 37 °C water bath. After removing the freezing medium through centrifugation, cell pellets were resuspended and divided, where half seeded at physiological density onto FNC-coated glass coverslip to assess cellular attachment, and were maintained in M5-Endo for several days. The remaining half was placed in a solution containing Fluorescein isothiocyanate (FITC) Annexin V apoptosis detection kit with Propidium Iodide (BioLegend, San Diego, CA, USA) following the manufacturer’s instructions, and analysed via flow cytometry for total live cells using a FACS Verse flow cytometer (Becton Dickinson, East Rutherford, NJ, USA) as previously described^19^.

### Gene expression analysis

Total RNA of confluent CEnCs from either the second or third passage was extracted using the Qiagen RNeasy kit (Qiagen, Hilden, Germany) and purified using Turbo DNA-free™ DNase treatment (Life Technologies). Reverse transcription of the RNA to cDNA was achieved using the SuperScript First-Strand Synthesis System (Life Technologies). Quantitative PCR was carried out on a Lightcycler 480 system (Roche), with 45 cycles of DNA denaturation (95 °C; 10 s), annealing (60 °C; 15 s), and extension (72 °C; 30 s), using Maxima SYBR green qPCR master mix (Thermo Scientific). Samples were run in triplicate, and the gene expression levels were normalized with the endogenous levels of glucose 6-phosphate dehydrogenase (GAPDH), and relative fold changes were analyzed using the comparative CT method. Primers used in this study were as follows (5′-3′): collagen type VIII alpha 2 chain (COL8A2), forward - GGGCAAAGGCCAGTACCT and reverse - GGGCTCTCCCTTCAGGTC; solute carrier family 4 member 11 (SLC4A11), forward - TGCTCTATGGCCTCTTCCTC and reverse - CCCTCCGGATGTAGTGTGTC; Glypican 4 (GPC4), forward - CCCTCCACGAGATCAACG and reverse - CTTTGCAGGCTGTACTTCTCC; OX-2 membrane glycoprotein (CD200) forward - AGACCACCATCAATGATTACCA and reverse - ACCACTGCTGCCATGACC; aldehyde dehydrogenase 3 family member A1 (ALDH3A1) forward - CATTGGCACCTGGAACTACC and reverse - GGCTTGAGGACCACTGAGTT; and Lumican (LUM) forward - CCTGGTTGAGCTGGATCTGT and reverse -TGGTTTCTGAGATGCGATTG.

### Immunocytochemistry

Confluent human CEnCs_(GMP)_ from the second passage were dissociated and plated at a density of at least 2,000 cells per mm^2^ on Collagen IV-coated glass coverslips and maintained for approximately seven days in M5-Endo before fixation with the appropriate fixative (Table 2). Samples were rinsed and blocked in 5% normal goat serum in PBS for 30 min at room temperature. Subsequently, samples were incubated with the primary antibodies at room temperature for 1 hour or at 4°C overnight. Primary antibodies used in this study are as listed in Table 2. The samples were then washed twice with PBS, 5 min each, and labeled with AlexaFluor 488 conjugated goat anti-mouse IgG secondary antibody (2.5 µg/ml, Life Technology) for 1 hour at room temperature in the dark. After two brief PBS washes, samples were mounted in Vectashield containing DAPI (Vector Laboratories, Burlingame, CA, USA), and visualized using a Zeiss Axioplan 2 fluorescence microscope (Carl Zeiss, Oberkochen, Germany).

**Table 2:** Information of primary antibodies used in the study.

Antibody (Clone)	Company (Catalog Number)	Fixative	Concentration
Na^+^/K^+^-ATPase (0.T.1)	Santa Cruz (sc-71638)	100% Ethanol 5 minutes, 4 °C	5 µg/mL
ZO-1 (1/ZO-1)	BD Pharmingen (610966)	100% Ethanol 5 minutes, 4 °C	5 µg/mL
CD-166 (TAG-1A3)	In-house^29^	100% Ethanol 5 minutes, 4 °C	Supernatant
PRDX-6 (TAG-2A12)	In-house^29^	Fix and Perm (Medium A) 15 minutes, room temp.	Supernatant
Human Nuclei (235-1)	Merck (Chemicon) (MAB1281)	4% Paraformaldehyde 10 minutes, 4 °C	1:100 dilution

The three commercially available primary antibodies used in this study, Na^+^/K^+^-ATPase, ZO-1 and Human Nuclei are listed here by their clone name, the company of which the primary antibody was purchased from, with their respective catalog numbers. Both CD-166 (TAG-1A3) and PRDX-6 (TAG-2A12) are generated in-house as described^29^. The different methods of fixation and concentration used were optimized for the staining of primary human CEnCs.

### Karyotype analysis

Karyotyping was performed by Singhealth Cytogenetics Laboratory, Department of Pathology (Singapore) using standard protocols for high-resolution Giemsa banding on metaphases obtained from cultures of human CEnCs_(GMP)_ at the third passage. A minimum of 20 metaphases from at least two cultures were analyzed for each donor. Samples with less than 20 metaphase obtained were excluded. A sample was considered ‘abnormal’ if the identified chromosomal aberrations can be deemed to be clonal, such that a minimum of two metaphase cells with the same trisomy of identical structural aberration or with the same structural abnormality, or if it possessed at least three cells which are monosomic for the same missing chromosome.

### Preparation of tissue-engineered corneal endothelial graft

Separate series of donor corneas deemed unsuitable for both transplantation and endothelial cell culture were prepared as construct carriers for the generation of TE-EK graft material. These donor corneas were generally either older in term of donor age (>40 years), or days of preservation (>14 days), as well as being of low endothelial cell count (<2,000 cells). Laser dissection of a layer of ultrathin human corneal stroma lenticules with DM-endothelium complex intact, at a diameter of at least 8.0 mm, was performed using a Ziemer laser (LDV) femtosecond laser system as described^65^. Corneoscleral buttons were dissected from the endothelial surface with the lamellar dissection depth set at 100 µm. Following the completion of the laser procedure, the DM/stroma lenticule was gently separated from the corneal button with a Chansue dissector, and carefully transferred, endothelial side-up, into a 4-well plate filled with sufficient PBS to keep the whole DM/stroma lenticule submerged. Subsequently, the corneal endothelial cells on the DM/stroma lenticule were gently denuded following three freeze/thaw cycles (−20 °C and room temperature). To determine that the denuded DM/stroma lenticules were devoid of donor endothelial cells, these lenticules were briefly treated with a Trypan Blue solution (0.1%) for 30 seconds and viewed under a stereoscopic dissecting microscope (Nikon, Toyko, Japan). The denuded DM/stroma lenticules were then left in M5-Endo medium overnight to ascertain that sterility was maintained, and subsequently stored at −20 °C until used.

One week before the scheduled transplantation, the denuded frozen DM/lenticules was taken out from frozen storage, thawed and trephined into a 6.0 mm circular disc with a 6.0 mm corneal trephine blade (Asico, Westmont, IL, USA). Primary human CEnCs at the first or second passage were dissociated and seeded onto the 6.0 mm DM/lenticule at a physiological density of 3,000 cells/mm^2^, and maintained in M5-Endo medium for approximately 5 to 7 days until the day of surgery. The constructed tissue-engineered corneal endothelial grafts were maintained in a humidified atmosphere at 37°C and 5% CO_2_, with medium refreshed every 2 days.

### Animal surgeries

Unless otherwise stated, all animal surgeries and follow-up evaluations were performed under general anesthesia achieved by intramuscular injections of xylazine hydrochloride (5 mg/kg; Troy Laboratories, Glendenning, NSW, Australia) and ketamine hydrochloride (50 mg/kg; Parnell Laboratories, Alexandria, NSW, Australia), together with a topical application of lignocaine hydrochloride (1%; Pfizer Laboratories, New York City, NY, USA).

### Cataract extraction surgery

For cataract extraction surgery of the rabbits performed by either YCL or MB, tropicamide (1%; Alcon Laboratories, Fort Worth, TX, United States) and phenylephrine hydrochloride (2.5%; Alcon Laboratories) eye drops were administered 30 minutes before the surgery to achieve mydriasis. A clear corneal incision was made with a 2.8 mm disposable keratome. After viscoelastic material containing sodium chondroitin sulphate and sodium hyaluronate (Viscoat; Alcon Laboratories) was instilled into the anterior chamber, a 5.0 mm diameter continuous curvilinear capsulotomy of the anterior capsule was created. Hydro-dissection was performed using a 27-gauge cannula. The lens was then aspirated and removed with a standard phacoemulsification procedure using the White Star Signature® phacoemulsification system (Abbott Medical Optics, Santa Ana, CA, USA). Subsequently, the corneal incision was sutured with 10/0 nylon suture and the rabbits were left aphakic with an intact posterior capsule for at least one week before TE-EK surgery.

### TE-EK surgery and follow-ups

All TE-EK surgeries performed were based on the EndoGlide insertion technique^57^ with minor modifications to accommodate the insertion of the tissue-engineered corneal endothelial graft into the anterior chamber of the rabbit. All TE-EK surgeries were performed by JSM. Briefly, a 4.5 mm temporal scleral tunnel main incision was made to facilitate TE-EK graft insertion with the EndoGlide. An anterior chamber maintainer was pre-placed to prevent the rabbit’s anterior chamber from collapsing during surgery. An approximate 7 mm of the rabbit’s DM was stripped and removed. A clear cornea paracentesis was made directly across from the temporal wound as the entry point of the curved EndoGlide placement forceps required to pull the engineered graft from the EndoGlide into the anterior chamber.

The endothelial cell surface of the prepared TE-EK graft was covered with a thin coat of dispersive viscoelastic before they were carefully loaded into the EndoGlide and inverted 180° prior to insertion, so that the endothelial cell surface faced downwards. The device was inserted into the temporal wound until the anterior opening of the EndoGlide was seen through the cornea to be fully within the anterior chamber. Moderate flow from the anterior chamber maintainer and the tight wound seal around the EndoGlide during the insertion process helps maintained a deep anterior chamber. With the EndoGlide in position, the placement forceps is inserted with the contralateral hand through the opposing paracentesis into the anterior chamber and over the glide surface of the EndoGlide. The leading edge of the TE-EK graft was grasped and pulled out of the EndoGlide and into the anterior chamber where it automatically uncoiled into the correct anatomic position with the endothelial surface facing down. While still holding the graft with the forceps, the EndoGlide was removed. Fully uncoiling of the inserted TE-EK graft may require gentle sideways or to-and-flow movements of the graft with the placement forceps. Once the TE-EK graft was fully uncoiled, an air bubble was injected beneath the inserted graft to float it against the stromal surface of the rabbit cornea. The main scleral wound and the anterior chamber maintainer paracentesis site were secured with 10/0 nylon interrupted sutures, the graft centered, and more air was injected for a full air tamponade of at least 6 minutes to facilitate graft adhesion to the posterior corneal surface of the rabbit cornea. Subsequently, some air was removed, leaving a smaller air bubble approximately the size of the TE-EK graft. The TE-EK procedure concluded with a 0.2 mL anti-inflammatory and anti-infective subconjunctival injection of a 1:1 mixture of dexamethasone sodium phosphate (4 mg/mL; Hospira, Melbourne, VIC, Australia) and gentamicin sulfate (40 mg/mL; Shin Poong Pharmaceutical, Seoul, Korea).

### Post-operative care

Following transplantation of the TE-EK graft, all rabbits received a post-operative regime of topical steroid prednisolone acetate (1%; Allergan Inc, Parsippany-Troy Hills, NJ, USA) and topical antibiotic tobramycin (1%; Alcon) for 4 times a day. An intramuscular injection of dexamethasone sodium phosphate (1 mL/kg; Norbrook Laboratories, Newry, Northern Ireland, UK) was also administered once daily. This medication regime was maintained for 28 days.

### Corneal imaging and intra-ocular pressure measurement

All corneal imaging and measurements of intra-ocular pressure (IOP) were performed prior to transplantation, at the fourth day, followed by 1, 2, 3 and 4 weeks after TE-EK surgery. Slit lamp photographs were taken with a Zoom Slit Lamp NS-2D (Righton, Tokyo, Japan) and AS-OCT scans of corneal cross-sections and measurements of corneal thickness were performed using a Visante AS-OCT (Carl Zeiss Meditec, Dublin, CA, USA). Three measurements were taken for the assessment of central corneal thickness, at the center (0.0 mm), and at 1 mm either side of the center (+1.0 mm, and −1.0 mm), and the mean value reported. Measurements of IOP were measured using a calibrated tonometer (Tono-pen Avia Vet; Reichert Ophthalmic Instruments, Depew, NY, USA).

The corneal endothelial cell density of the transplanted TE-EK graft was obtained from *in-vivo* confocal images taken using the Heidelberg Retina Tomography (HRT) 3 system in combination with the Rostock Corneal Module (Heidelberg Engineering GmbH, Heidelberg, Germany). At least three confocal images were taken at Day 28 and a region of interest of between 50 to 100 cells was selected for cell density assessment using standard software available within the HRT system.

### Analysis of TE-EK graft

The rabbits were euthanized under anesthesia at 4 weeks by an overdose intracardiac injection of sodium pentobarbitone (85 mg/kg; Jurox, Rutherford, NSW, Australia). The corneas were excised from the globe, halved and subjected to the respective processes detailed below for analysis.

### Immunohistochemistry

For immunohistochemistry, excised corneal sample was embedded in a frozen section compound (Surgipath; Leica Microsystems, Nussloch, Germany), and stored at −80 °C until sectioning. Serial sections of 10 µm sections were cut using a HM525 NX cryostat (Thermo Scientific, Waltham, MA, USA). The sections were collected on polylysin-coated glass slides (Thermo Scientific), and labelled using a mouse IgG1 anti-human nuclei antibody (Table 2) based on the immunocytochemistry approach as described earlier.

### Histochemistry – trypan blue and alizarin red staining

For histochemistry, each excised corneal sample was first placed endothelial side up and first stained for 3 minutes in a buffered Trypan blue solution (0.2%). The excess was decanted and the cornea was the subsequently stained in freshly prepared and filtered Alizarin red solution (0.5%; pH 4.5). The stained specimen was then washed for 60 seconds in a wash buffer, prior to wet mounting and examined using a Zeiss Axioplan 2 microscope.

### Scanning electron microscopy

Excised TE-EK graft specimens for scanning electron microscopy were first immersed overnight in a fixative solution consisting of 2% glutaraldehyde in PBS (pH 7.4; Electron Microscopy Sciences, Hatfield, PA, USA) at 4 °C overnight. Fixed specimen was washed thrice in PBS for 5 minutes each, and kept in 1% osmium tetroxide at room temperature for 1 hour. The sample was then dehydrated in an increasing concentration of ethanol at 25%, 50%, 75%, 95% and 100% for 10 minutes each, with the last step repeated thrice. Dehydrated samples were then dried in a critical point dryer (BALTEC, Balzer, Liechtenstein), and mounted onto a SEM stub using carbon adhesive tabs. Samples were then sputter-coated with a 10 nm layer of gold (BALTEC), and examined under a scanning electron microscope (Quanta 650FEG; FEI, Hillsboro, OR, USA).

### Statistics

All numeric data obtained were expressed as mean ± standard deviation (SD). All statistical analyses were performed using SPSS Statistics 22.0 (IBM, Chicago, IL, USA). The two comparisons of cellular attachment of human CEnCs on various extra-cellular matrices (Fig. 2A), and the corneal thickness of TE-EK (Fig. 4A) were performed using one-way ANOVA followed by post-hoc Bonferroni test for multiple comparisons. All results with a *p*-value of less than 0.05 were deemed to be statistically significant.

## Electronic supplementary material

Supplementary Information
