# Supplementary material for: Regulatory Compliant Tissue-Engineered Human Corneal Endothelial Grafts Restore Corneal Function of Rabbits with Bullous Keratopathy
Source: Sci Rep. 2017 Oct 26;7:14149. doi: 10.1038/s41598-017-14723-z (PMC5658403; doi:10.1038/s41598-017-14723-z)
Supplement: Supplementary file 1 — Supplementary Information [file 41598_2017_14723_MOESM1_ESM.pdf]

Supplementary Information

Regulatory Compliant Tissue-Engineered Human Corneal Endothelial Grafts Restore Corneal Function of Rabbits with Bullous Keratopathy

Gary S. L. Peh[1, 2], Heng-Pei Ang[1], Chan N. Lwin[1], Khadijah Adnan[1], Benjamin L. George[1,2], Xin-Yi Seah[1], Shu-Jun Lin[1], Maninder Bhogal[1,6], Yu-Chi Liu[1,3], Donald T. Tan[1,3,5], Jodhbir S. Mehta[1,2,3,4]**

[1]Tissue Engineering and Stem Cell Group, Singapore Eye Research Institute, Singapore.

[2]Duke-NUS Graduate Medical School, Singapore.

[3]Singapore National Eye Centre, Singapore.

[4]School of Material Science and Engineering, Nanyang Technological University, Singapore.

[5]Yong Loo Lin School of Medicine, National University of Singapore, Singapore.

[6]Department of Corneal and External Disease, Moorfields Eye Hospital, London, UK.

*To whom correspondence should be addressed: garypeh@gmail.com or jodmehta@gmail.com

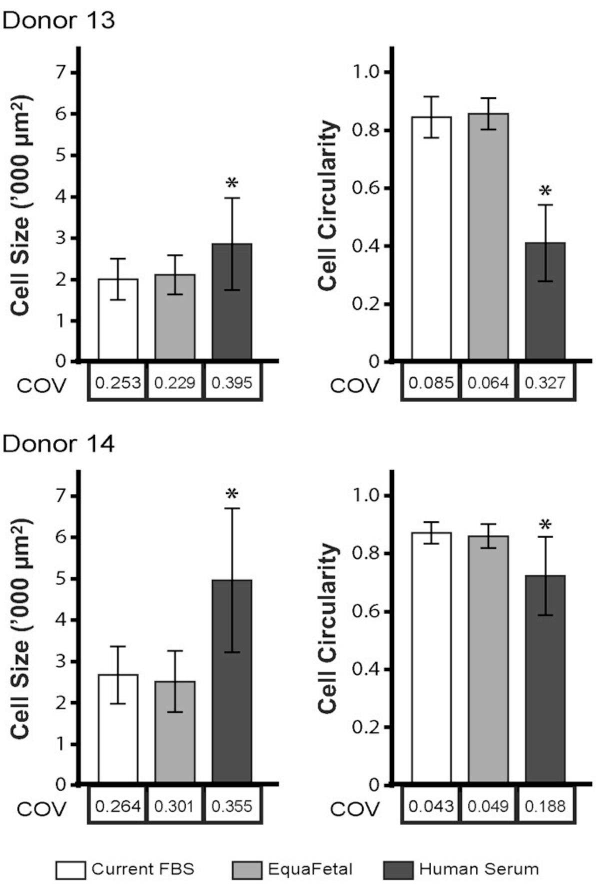

Supplementary Figure S1 | Morphometric analysis of donor-matched CEnCs grown in different serum. Cell size, cell circularity and their respective coefficient of variance (COV) were analyzed for two donors. For both donors, CEnCs expanded in the dual media supplemented with either regular FBS or EquaFetal were comparable, whereas CEnCs cultured in HS supplemented media were significantly larger in size and significantly less circular, indicating that they were more elongated in shape, similar to fibroblast-like cells, and were also found to have a greater degree of variability with higher COV values in both cell size and cell circularity. Between the two donors evaluated, CEnCs cultured in HS supplemented media also showed higher degree of inconsistency in terms of both cell size and cell circularity.

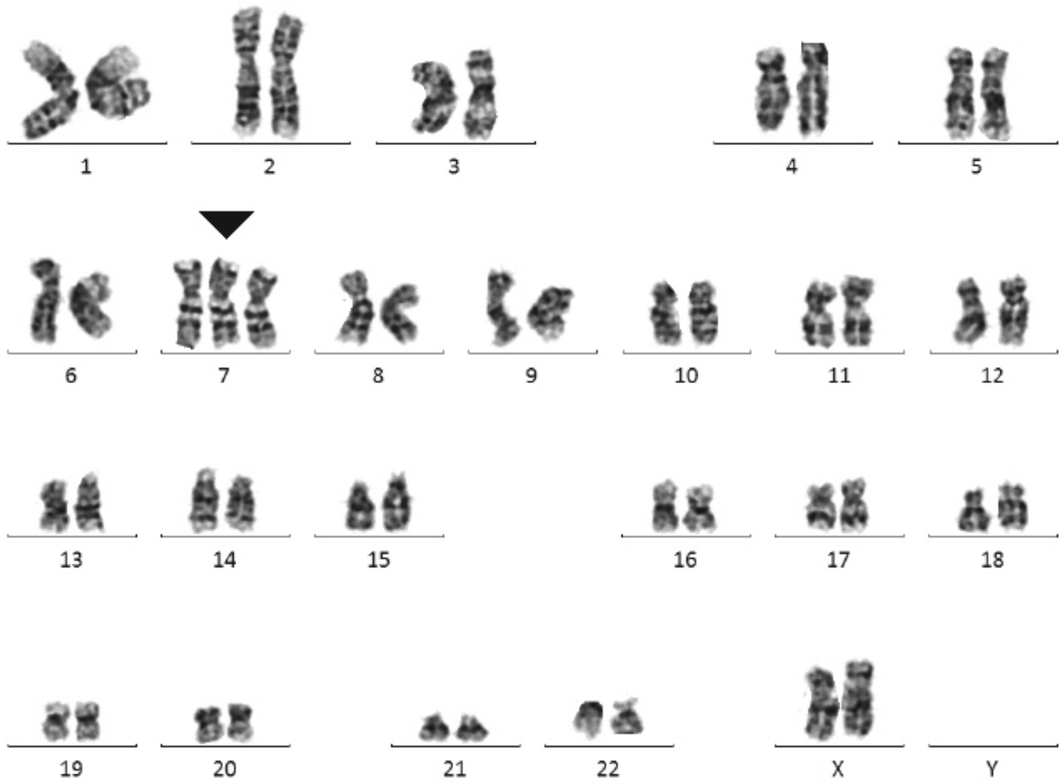

Supplementary Figure S2 | Karyogram of Trisomy 7 detected in cultured CEnCs. Chromosomal aberration of chromosome 7 trisomy (47, XX +7; arrowed) detected in 1 out of the 4 cultures of CEnCs assessed.

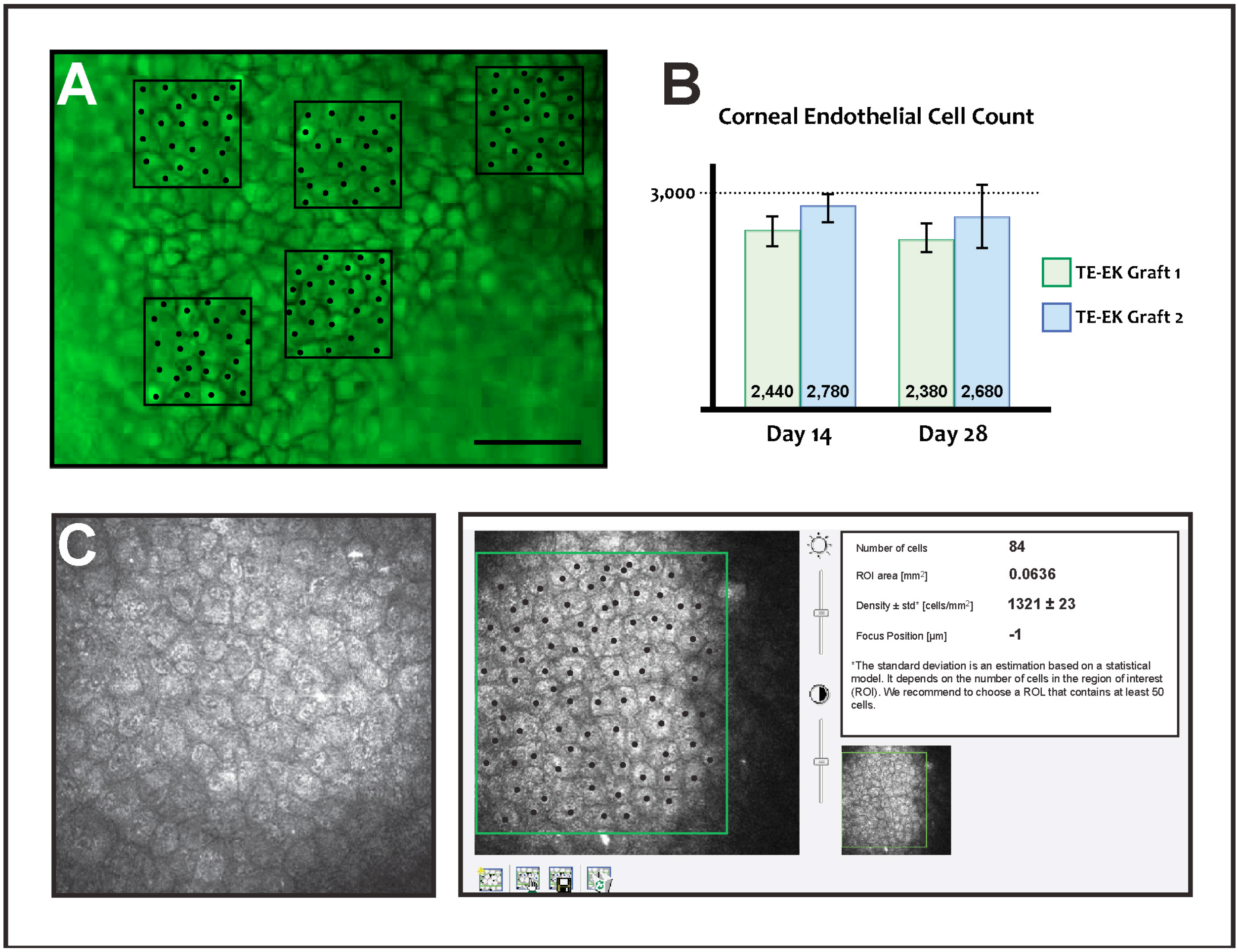

Supplementary Figure S3 | Corneal endothelial cell count of TE-EK grafts. (A) Representing image of the TE-EK graft labeled with Calcein AM, where overall cell counts were obtained through extrapolation of 5 randomly selected 100µm x 100µm areas that were manually counted. Scale bar: 100µm. (B) Cell counts obtained for TE-EK graft 1 was 2,440 ± 207 at Day 14 and 2,380 ± 192 at Day 28; and for TE-EK graft 2 was 2,780 ± 179 at Day 14 and 2,680 ± 438 at Day 28. (C) Representative confocal image of the transplanted TE-EK graft taken at Day 28 using the Heidelberg Retina Tomography system (left), and a representative screen capture of the corneal endothelial cell density of 1,321 ± 23 cells obtained using standard software available within the HRT system.

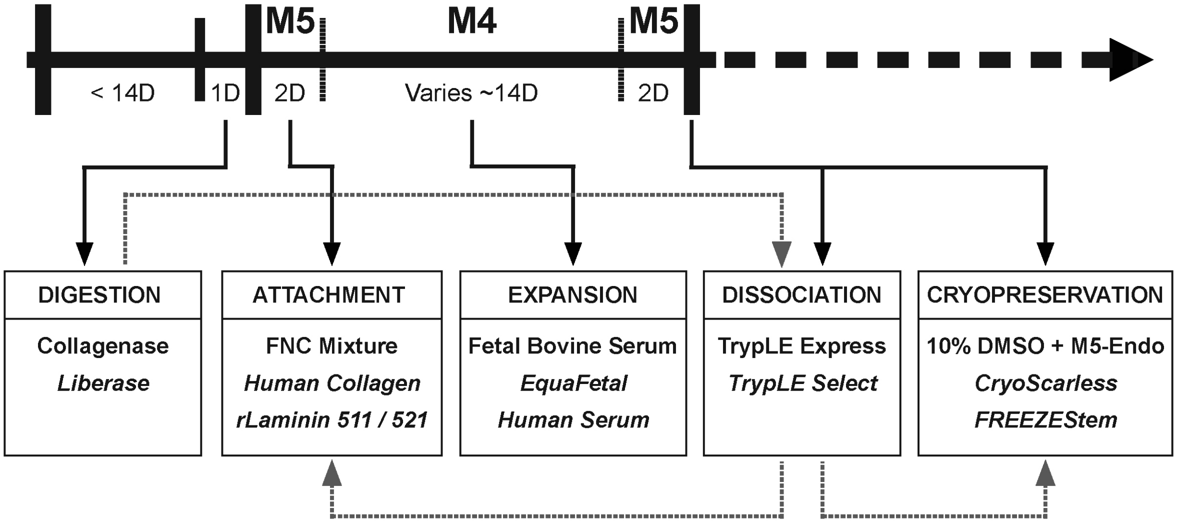

Supplementary Figure S4 | Schematic figure of the dual media culture system and the initial assessment of reagents. The schematic illustrated the various key processes that were assessed in this study to better define the culture reagents used. These processes were specific to cellular digestion and dissociation during isolation; cellular attachment following isolation; proliferation dynamics during cell expansion; and cell dissociation for sub-culture of the CEnCs. Cryo-preservation of the human CEnCs was also assessed.

Supplementary Table S1. Donor information for DM/stromal lenticules used in the study			
**Serial Number**	**Age**	**Sex**	**Cause of Death**
01	58	M	Multi-System Organ Failure
02			
03	25	M	Multi-Vehicle Accident
04	47	F	Cancer – Cervix
05	51	M	Cardiomegaly
06	69	M	Metastatic Cancer
07	68	M	Cancer – Liver
08	51	M	Metastatic Cancer
09	40	F	Pulmonary Thromboembolism
10	69	M	Severe Malnutrition
11			

Supplementary Table S1. Donor corneas procured were processed and used for the generation of the DM/stroma lenticular discs using the Ziemer laser Doppler velocimetry (LDV) femtosecond laser system. These lenticular discs were then denuded and further processed as the biological stromal carrier with DM intact used in the creation of the TE-EK graft material.
